# Influence of the Competitive Level and Weight Class on Technical Performance and Physiological and Psychophysiological Responses during Simulated Mixed Martial Arts Fights: A Preliminary Study

**DOI:** 10.5114/jhk/159453

**Published:** 2023-01-20

**Authors:** Orlando Folhes, Víctor Machado Reis, Diogo Luís Marques, Henrique Pereira Neiva, Mário Cardoso Marques

**Affiliations:** 1 Department of Sport Sciences, University of Beira Interior, 6201-001 Covilhã, Portugal.; 2 Department of Sport Sciences, Exercise and Health, University of Trás-os-Montes e Alto Douro, 5001-801 Vila Real, Portugal.; 3 Research Center in Sports Sciences, Health Sciences and Human Development, CIDESD, 6201-001 Covilhã, Portugal.

**Keywords:** combat sports, fight simulation, hemodynamics, rate of perceived exertion

## Abstract

This study aimed to analyze the influence of the competitive level and weight class on technical performance and physiological and psychophysiological responses during simulated MMA fights. Twenty MMA male athletes were divided into four groups: heavyweight elite (HWE; n = 6), lightweight elite (LWE; n = 3), heavyweight professional (HWP; n = 4), and lightweight professional (LWP; n = 7). All athletes performed four simulated fights of three 5-min rounds with a 1-min rest interval. Each fight was recorded using a video camera to analyze offensive and defensive actions. Moreover, the following measures were made: heart rate (before and after each round), blood lactate concentration (before and after the fight), readiness state (before each round), and the rate of perceived exertion (RPE) (after each round). The main findings were: i) LWE athletes applied more offensive touches than LWP athletes; ii) HWP athletes presented higher heart rate values than LWP athletes after the first round; however, LWP athletes presented greater heart rate changes than HWP athletes from the first to the second round; iii) no differences existed between groups in blood lactate concentration and readiness state; and iv) HWP and LWP athletes presented higher RPE values than LWE athletes in the first and third rounds; however, LWE athletes presented greater RPE changes than HWE, HWP, and LWP athletes from the first to the second and third rounds. This study shows that LWE athletes apply more offensive touches than LWP athletes during simulated MMA fights. Moreover, lightweight athletes tend to increase their physiological demand as the combat evolves, which is also reflected in their RPE.

## Introduction

Mixed Martial Arts (MMA) training includes technical, physical, and tactical development sessions. Most sessions involve direct physical contact with an opponent simulating actions possible to find in an official combat (Barley and Harms, 2021). In combat, athletes execute a wide range of motor actions in attack and defense within a time largely standardized in three rounds of 5 min each, with a 1-min rest interval in between (Tota et al., 2019).

Lachlan et al. (2013) stated that MMA combats incorporate striking and grappling techniques. Athletes can use punches, kicks, knees, and elbows to the head, face, and body as offensive techniques, grabbing the opponent, trying to gain control of the opponent's body, and projecting it into the ground fight. Once on the ground, they can try to gain dominant positions to apply chokes or twists or even try to get back on their feet. Thus, the variety of motor actions is tremendous, and their combinations turn out unpredictable when in a combat situation.

The availability of data on physiological responses resulting from MMA matches has been increasing, but it is still quite limited (Marinho et al., 2016; Schick et al., 2010; Soncin, 2016) compared to modalities such as judo and wrestling (Alm, 2013; Amtmann et al., 2008). Del Vecchio et al. (2011) described MMA as a combat modality with high energy demand interspersed with high-intensity actions, emphasizing the glycolytic system. On the other hand, it has been shown that body composition may affect the glycolytic energetics during simulated Jiu-Jitsu combats (Pessôa Filho et al., 2021). In MMA, athletes are grouped by the weight class, as in other combat sports (Faro et al., 2022). In different weight classes, fights can have different technical and physical profiles. Typically, it is observed that in lower weight classes, actions are faster and more frequent. Nevertheless, to our knowledge, no study has examined technical and physiological factors that differentiate between MMA athletes of different weight classes and competitive levels during the fight. Therefore, with the popularization and an exponential increase in the number of MMA athletes versus the limited amount of information in the scientific literature on the physiological characteristics of the modality, it is necessary to understand physiological responses and technical behavior during a simulated MMA fight.

Therefore, the current study aimed to verify the influence of the competitive level and weight class on technical performance and physiological and psychophysiological responses during simulated MMA fights.

## Methods

### Study Design

**Figure 1:**
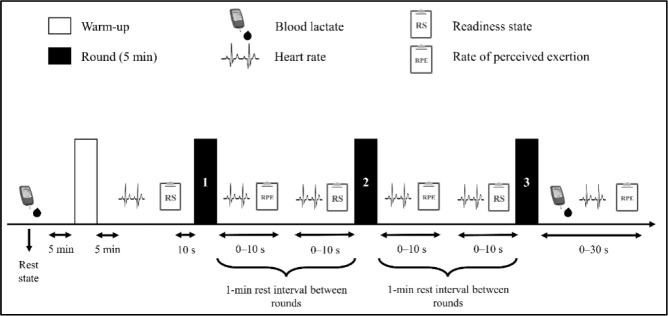
Illustration of the experimental procedures.

This is a cross-sectional study, in which professional and elite Brazilian MMA athletes volunteered to perform four simulated MMA fights for two weeks (two fights per week) with a 72-h recovery between fights. Athletes were grouped by their weight class for the fights, and the pairs were drawn. Athletes were not previously informed about the identity or strategy used by the opponents not to influence the fight development. Regardless of a fight simulation, the time and rules were the same as for a legal fight, and athletes were allowed to use extra protections (e.g., shin pads, knee pads, elbow pads, and helmets). Every fight was recorded using a video camera (SONY DCRSX22, São Paulo, Brazil) attached to a tripod to assess technical performance. Moreover, the heart rate was measured before and after each round, while blood lactate concentration was measured before (at rest) and after fights. Finally, the readiness state was evaluated before each round, while the rate of perceived exertion (RPE) was measured after each round. All athletes were advised to maintain their regular hydration and nutrition routines and avoid strenuous exercise 72 h before the simulated fights. Figure 1 illustrates the study design.

### Participants

Twenty Brazilian male MMA athletes were divided into four groups according to their weight class and competitive level: heavyweight elite (*n* = 6; 33.8 ± 2.5 years; 81.4 ± 5.1 kg; 1.8 ± 0.1 m), heavyweight professional (*n* = 4; 36.5 ± 2.4 years; 80.8 ± 3.4 kg; 1.8 ± 0.0 m), lightweight elite (*n* = 3; 33.0 ± 2.0 years; 69.5 ± 6.6 kg; 1.7 ± 0.1 m), and lightweight professional (*n* = 7; 30.9 ± 4.2 years; 71.7 ± 4.7 kg; 1.7 ± 0.1 m). Athletes had more than ten years of combat experience and were considered professional when they participated in more than three professional MMA fights in events accredited by the “Comissão Atlética Brasileira de MMA” (CABMMA), including the UFC, BELLATOR, ONE FC, and SHOOTO. Briefly, UFC, BELLATOR, and ONE FC consist of five 5-min rounds for championship fights and three 5-min rounds for non-title bouts. SHOOTO pro-fights consist of three 5-min rounds, while semi-pros fights consist of two 5-min rounds. If athletes were ranked among the top ten in their categories based on these events, they were rated as elite MMA athletes. Athletes weighing ≤ 76 kg were considered lightweight, and those weighing > 76 were considered heavyweight, according to CABMMA unified rules (CABMMA, 2019). Before starting the study, we informed the coaches and athletes about the experimental procedures. The Review Board of the Department of Sport Sciences at the University of Beira Interior (project D1942, July 2017) approved this study, and all athletes signed a written informed consent form according to the Declaration of Helsinki.

### Simulated MMA Fights

**Figure 2:**
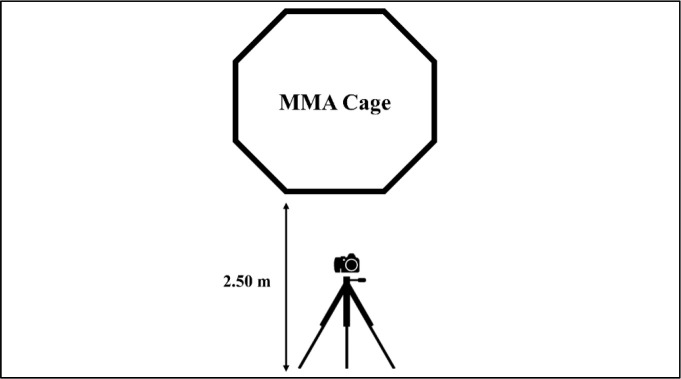
Illustration of the position of the video camera.

Before each fight, athletes performed a warm-up similar to that in official competitions. The warm-up lasted 30 min and included joint mobility exercises, followed by non-specific movements for neuromuscular activation and a specific warm-up with fight movements. Each fight consisted of three 5-min rounds with a 1-min rest interval in between. In the event of submission by strangulation or key, the fight was restarted until the end. In knockout cases, the fight was interrupted and excluded from the analysis. There was no referee inside the cage. Instead, a health practitioner and a certified referee watched the fights outside the cage. Only in the event of a serious injury risk or a fight-ending sequence would they interrupt. The video camera was positioned 2.50 m away from the MMA cage and attached to a stationary tripod, as shown in Figure 2. The video recordings of each fight were edited using Wondershare Filmora v9 software (Wondershare Software Co., Ltd., South Shenzhen, China). In each video, the following variables were evaluated: i) the number of touches (including punches, kicks, knees, and elbows), ii) the number of projections, and iii) the number of submissions suffered and applied by each athlete. The average value of the four simulated fights was used to analyze each variable.

### Heart Rate, Blood Lactate Concentration, Readiness State, and the Rate of Perceived Exertion

The heart rate was measured using a monitor (Polar FT7F, Kempele, Finland) connected via Bluetooth to a Polar watch and attached below the chest. Next, blood lactate concentration was measured using a handheld lactate analyzer (Accutrend Plus Roche, Rio de Janeiro, Brazil). After cleansing the site with 70% alcohol, the ear lobe was punctured using a disposable lancet (Accu-Chek Aviva Test Strips). The first drop of blood was discarded to avoid contamination with sweat, and then a blood sample collected in an assay strip was inserted into the device. The readiness state was measured by asking athletes if they were ready to start the rounds, and two possible answers were provided: “yes” or “no”. Finally, the RPE was measured using the Borg 0– 10 scale, which ranges from 0 (no exertion) to 10 (maximum exertion) (Borg, 1982). The average value of the four simulated fights was used for further analysis.

### Statistical Analysis

Data are presented as means and standard deviations with 95% confidence intervals. The percent changes from rounds 1 to 2, 2 to 3, and 1 to 3 in the heart rate and the RPE were calculated. In addition, the percent change from pre- to post-fights in blood lactate concentration was calculated. The Shapiro-Wilk test checked the normality of each variable. Since some variables violated the normality assumption, parametric and non-parametric tests were used. One-way ANOVA or Kruskal-Wallis tests analyzed the differences between groups in offensive and defensive actions, the heart rate, the RPE, and blood lactate concentration. If significant differences were detected, Bonferroni post hoc tests were conducted. The Pearson's chi-squared test compared the differences between groups in the readiness state. All statistical analyses were conducted in Microsoft Office Excel^®^ (Microsoft Inc., Redmond, WA, USA) and SPSS v27 (SPSS Inc., Chicago, IL, USA), and the alpha level was set at *p* < 0.05.

## Results

### Technical Performance Differences between Groups

Table 1 shows differences between groups in offensive touches, with the post hoc tests revealing a higher number of touches of LWE than LWP athletes. For the remaining variables, there were no differences between groups (*p* > 0.05).

**Table 1:** Differences between groups in offensive and defensive actions.

	HWE (*n* = 6)		LWE (*n* = 3)	HWP (*n* = 4)	LWP (*n* = 7)
**Offensive Touches**					
Mean ± SD	43.2 ± 17.1		60.3 ± 23.6	32.4 ± 4.1	23.8 ± 8.2
95% CI	29.5–56.9		33.5–87.0	28.3–36.4	17.7–29.8
*p* between ^a^	0.02				
Post hoc ^b^	LWE > LWP (*p* = 0.03)				
**Offensive Finishing**					
Mean ± SD	0.4 ± 0.3	0.8 ± 1.3		1.5 ± 0.8	1.3 ± 1.1
95% CI	0.1–0.7	-0.7–2.2		0.7–2.3	0.5–2.1
*p* between ^a^	0.20				
Post hoc ^b^	NA				
		**Offensive Projections**			
Mean ± SD	4.1 ± 4.0	2.3 ± 2.3		4.3 ± 0.2	3.3 ± 1.9
95% CI	0.9–7.3	−0.2–4.9		4.0–4.5	1.9–4.7
*p* between ^a^	0.84				
Post hoc ^b^	NA				
		**Defensive Touches**			
Mean ± SD	35.3 ± 17.5	40.6 ± 14.1		30.6 ± 10.5	29.4 ± 6.7
95% CI	21.4–49.3	24.6–56.5		20.3–40.9	24.4–34.3
*p* between ^a^	0.59				
Post hoc ^b^	NA				
		**Defensive Finishing**			
Mean ± SD	0.6 ± 0.6	0.2 ± 0.3		0.5 ± 0.6	0.8 ± 0.7
95% CI	0.1–1.1	−0.2–0.5		−0.1–1.1	0.2–1.3
*p* between ^a^	0.55				
Post hoc ^b^	NA				
		**Defensive Projections**			
Mean ± SD	2.1 ± 1.3	1.9 ± 1.9		3.2 ± 0.7	3.0 ± 1.2
95% CI	1.1–3.1	−0.2–4.0		2.5–3.8	2.2–3.9
*p* between ^c^	0.34				
Post hoc ^d^	NA				

CI: confidence interval; SD: standard deviation; HWE: heavyweight elite; HWP: heavyweight professional; LWE: lightweight elite; LWP: lightweight professional; NA: not applicable; ^a^ Kruskal-Wallis test; ^b^ Bonferroni correction for multiple tests; ^c^ One-Way ANOVA; ^d^ Bonferroni post hoc tests.

### Physiological Differences between Groups

**Figure 3:**
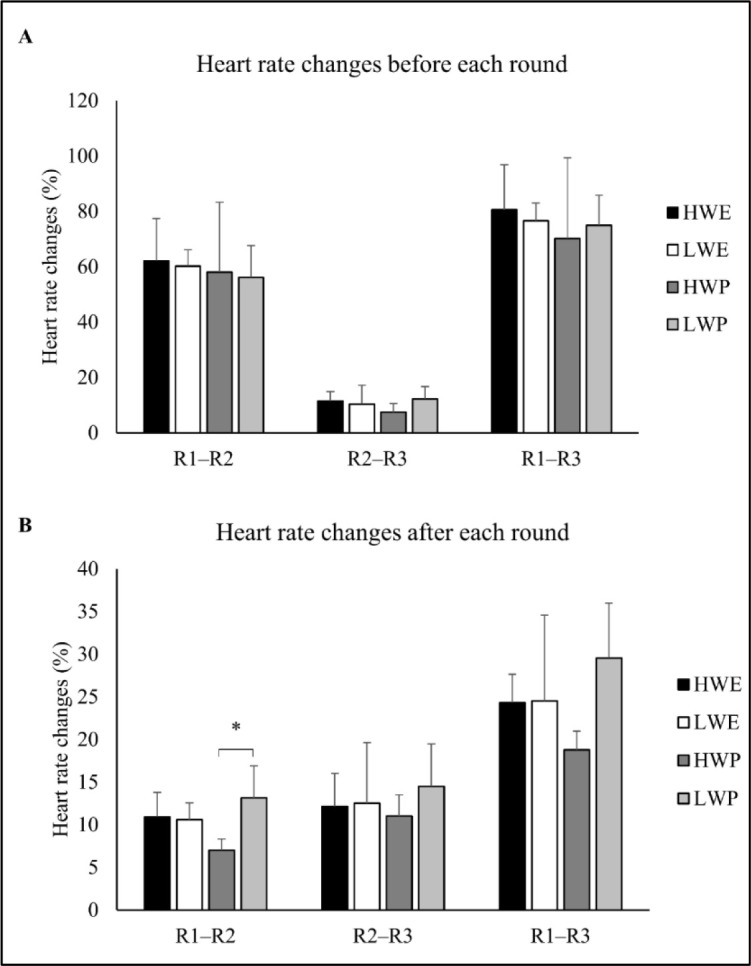
Heart rate changes from rounds 1 to 2, 2 to 3, and 1 to 3 before (A) and after (B) each round; * *p* < 0.05, significant differences between groups (one-way ANOVA); R: round; HWE: heavyweight elite athletes; HWP: heavyweight professional athletes; LWE: lightweight elite athletes; LWP: lightweight professional athletes.

Table 2 indicates differences between groups in the heart rate after the first round, with the post hoc tests showing a non-significant higher increase in HWP than LWP athletes. For the remaining rounds, there were no differences between groups (*p* > 0.05). Figure 3 shows differences between groups in heart rate changes (after the round) from rounds 1 to 2 (*p* = 0.03), with the post hoc tests revealing greater changes in LWP than HWP athletes (*p* = 0.02).

**Table 2:** Differences between groups in the heart rate before and after each round.

	Round 1		Round 2		Round 3	
	Before	After	Before	After	Before	After
**HWE**						
Mean ± SD	71.8 ± 6.8	145.5 ± 11.2	116.2 ± 14.1	161.5 ± 14.2	129.2 ± 12.6	181.1 ± 17.1
95% CI	66.3–77.2	136.5–154.5	104.9–127.5	150.1–172.8	119.1–139.2	167.4–194.8
**LWE**						
Mean ± SD	69.1 ± 6.4	141.3 ± 13.4	110.8 ± 11.8	156.1 ± 12.8	121.8 ± 6.8	175.4 ± 14.8
95% CI	61.9–76.3	126.1–156.4	97.4–124.1	141.5–170.6	114.0–129.5	158.7–192.2
**HWP**						
Mean ± SD	83.1 ± 11.9	161.8 ± 3.2	129.3 ± 6.2	173.1 ± 3.9	138.9 ± 7.9	192.1 ± 1.4
95% CI	71.4–94.8	158.6–164.9	123.2–135.3	169.3–176.9	131.2–146.6	190.7–193.4
**LWP**						
Mean ± SD	74.2 ± 4.7	143.6 ± 10.3	115.7 ± 8.9	162.3 ± 9.8	129.5 ± 6.3	185.5 ± 5.9
95% CI	70.8–77.6	136.0–151.2	109.1–122.2	155.0–169.5	124.9–134.2	181.1–189.8
***p between***	0.13 ^a^	0.04 ^b^	0.14 ^b^	0.24 ^b^	0.13 ^b^	0.13 ^a^
***Post hoc*** **(*p*-value)**	-	HWP > LWP (0.07 ^c^)	-	-	-	-

CI: confidence interval; SD: standard deviation; HWE: heavyweight elite athletes; HWP: heavyweight professional athletes; LWE: lightweight elite athletes; LWP: lightweight professional athletes; ^a^ Kruskal-Wallis test; ^b^ OneWay ANOVA; ^c^ Bonferroni post hoc tests.

Table 3 shows no differences between groups in blood lactate concentration before and after fights and in percent change (*p* > 0.05).

**Table 3:** Differences between groups in blood lactate concentrations.

	HWE	LWE	HWP	LWP	*p between*	*Post hoc*
**Before**						
Mean ± SD	1.9 ± 0.4	2.1 ± 0.7	2.4 ± 0.2	2.3 ± 0.4	0.17 ^a^	-
95% CI	1.6–2.1	1.3–2.9	2.3–2.6	2.0–2.7		
**After**						
Mean ± SD	9.5 ± 1.4	8.6 ± 0.8	9.9 ± 1.4	11.1 ± 1.5	0.08 ^a^	-
95% CI	8.3–10.6	7.6–9.5	8.6–11.3	10.0–12.1		
**%Δ**						
Mean ± SD	416.7 ± 40.1	327.2 ± 119.1	312.2 ± 53.6	381.8 ± 46.7	0.06 ^a^	-
95% CI	384.6–448.9	192.5–462.0	259.7–364.8	347.2–416.3		

CI: confidence interval; SD: standard deviation; HWE: heavyweight elite athletes; HWP: heavyweight professional athletes; LWE: lightweight elite athletes; LWP: lightweight professional athletes; %Δ: percent change from pre- to post-fights; ^a^ One-Way ANOVA.

### Psychophysiological Differences between Groups

There were no differences between groups in the readiness state at the beginning of each round, as all athletes (100%) provided an affirmative answer (i.e., “yes”).

Table 4 shows differences between groups in the RPE after rounds 1 and 2. Post hoc tests indicated that HWP and LWP athletes presented higher RPE levels than LWE athletes after rounds 1 and 2. In addition, Table 4 indicates differences between groups in RPE changes, with LWE athletes presenting a greater change from rounds 1 to 2 and 1 to 3 than HWE, HWP, and LWP athletes.

**Table 4:** Differences between groups in the rate of perceived exertion after each round.

	Round 1	Round 2	Round 3	%Δ Round 1–2	%Δ Round 2–3	%Δ Round 1–3
**HWE**						
Mean ± SD	3.5 ± 0.7	5.5 ± 0.7	8.3 ± 0.7	58.9 ± 12.2	51.7 ± 9.6	141.5 ± 29.4
95% CI	2.9–4.1	4.9–6.1	7.7–8.9	49.1–68-7	44.0–59.4	118.0–165.0
**LWE**						
Mean ± SD	2.7 ± 0.4	5.2 ± 0.8	7.8 ± 0.8	93.5 ± 5.8	51.1 ± 12.2	192.1 ± 16.5
95% CI	2.2–3.1	4.3–6.1	6.9–8.6	87.0–100.1	37.3–65.0	173.4–210.7
**HWP**						
Mean ± SD	4.3 ± 0.5	6.2 ± 0.2	8.8 ± 0.4	44.6 ± 12.9	42.4 ± 3.6	105.7 ± 15.9
95% CI	3.8–4.8	6.0–6.4	8.4–9.2	32.0–57.6	38.9–45.9	90.2–121.3
**LWP**						
Mean ± SD	4.4 ± 0.6	6.3 ± 0.7	9.3 ± 0.8	43.2 ± 11.4	49.9 ± 11.9	114.0 ± 17.8
95% CI	3.9–4.8	5.7–6.8	8.7–9.9	34.7–51.6	58.7–41.0	100.8–127.2
***p between***	< 0.01 ^a^	0.07 ^a^	0.02 ^a^	< 0.001 ^a^	0.53 ^a^	< 0.001 ^a^
***Post hoc*** **(*p*-value)**	HWP>LWE (0.02 ^b^)	-	LWP>LWE (0.03 ^b^)	LWE>HWE (< 0.01 ^b^)	-	LWE>HWE (0.03 ^b^)
	LWP>LWE (< 0.01 ^b^)			LWE>HWP (< 0.001 ^b^)		LWE>HWP (< 0.001 ^b^)
				LWE>LWP (< 0.001 ^b^)		LWE>LWP (< 0.001 ^b^)

CI: confidence interval; SD: standard deviation; HWE: heavyweight elite athletes; HWP: heavyweight professional athletes; LWE: lightweight elite athletes; LWP: lightweight professional athletes; %Δ: percent change; ^a^ One-Way ANOVA; ^b^ Bonferroni post hoc tests.

## Discussion

### Main Findings

This study aimed to verify the influence of the competitive level and weight class on technical performance and physiological and psychophysiological responses during simulated MMA fights. The main findings were that i) LWE athletes applied more offensive touches than LWP athletes; ii) HWP athletes presented higher heart rate values than LWP athletes after the first round; however, LWP athletes presented greater heart rate changes than HWP athletes from the first to the second round; iii) no differences existed between groups in blood lactate concentration and readiness state; and iv) HWP and LWP athletes presented higher RPE values than LWE athletes in the first and third rounds; however, LWE athletes presented greater RPE changes than HWE, HWP, and LWP athletes from the first to the second and third rounds.

### Technical Performance

The current study observed that LWE athletes applied more offensive touches (i.e., punches, kicks, knees, and elbows) during the simulated fights than LWP athletes. These findings may be associated with the higher fighting experience and competitive level of elite than professional MMA athletes. Indeed, a common strategy for professional athletes is to save energy, especially in the early rounds, to improve their chances of winning the latter rounds. On the contrary, less experienced athletes start fights at full speed, thereby displaying a more significant number of strikes, which sometimes leads to early fatigue. Although evidence from studies is scarce on this matter (this might be the first study with simulated MMA fights), such anecdotal reports exist in the MMA community and warrant further research. As to differences between weight classes, the present study did not detect significant differences in technical performance between these groups, which agrees with what was found with elite judo athletes in studies by Calmet et al. (2010) and Franchini et al. (2005).

### Physiological Responses

This study observed that LWP athletes presented greater heart rate changes from the first to the second round than HWP athletes. However, as HWP athletes had already presented higher heart rate values than LWP athletes after the first round, these differences might be due to the fighting strategy used in the first and second rounds. LWP athletes probably adopted a less cardiovascular demanding strategy in the first round than HWP athletes, but as the fight evolved, they increased the fighting intensity, resulting in a higher heart rate increase. Moreover, athletes with lower weight likely possess a relatively higher aerobic capacity (VO_2max_), enabling them to use a more prominent fraction of their heart rate reserve during the fight. Indeed, Calmet et al. (2010) and Franchini et al. (2005) have shown that lightweight judo elite athletes performed better in VO_2max_ tests when compared with heavyweight athletes.

Regarding blood lactate concentration, although no differences were observed between groups, it is interesting to notice that the values measured before and after the fights are in line with the studies by Siqueira et al. (2016) (pre-fight: 2.6 mmol/L, post-fight: 11.5 mmol/L), Coswig et al. (2016) (pre-fight: 2.2 mmol/L; post-fight: 15.6 mmo/L), and Amtmann et al. (2008) (post-fight: 10.2 mmol/L). In the study by Amtmann et al. (2008), who measured blood lactate concentration after real professional MMA fights, the values reached 20.7 mmol/L, thus higher than those in the present study, as well as in studies with simulated fights (Alm, 2013; Amtmann, 2004). The absence of the competitive expression may cause an increase in the duration and the number of low-intensity actions, which can directly influence the temporality of actions (Coswig et al., 2016) and limit the increase in blood lactate concentration. Although the literature presents this blood lactate variation, it is suggested that MMA is a high-intensity modality with a significant participation of the glycolytic system, given the high blood lactate concentrations found at the end of each combat simulation. Furthermore, the study by Siqueira et al. (2016) reinforces the high anaerobic demand during combat, demonstrating that the trainability and development of this bioenergetic system are of fundamental importance in preparing athletes for fights.

### Psychophysiological Responses

Regardless of the competitive level and weight class, athletes gave an affirmative response about their readiness state before each round. These findings suggest that a 3-min rest interval between rounds may be sufficient for elite and professional athletes to mentally recover during simulated MMA fights. Nevertheless, it would be essential to determine if this pattern is maintained during official MMA fights (those with three rounds). Therefore, since no similar studies can be found in the literature, future research should analyze the differences between MMA athletes of different competitive levels and weight classes in the readiness state responses during official MMA fights.

The data showed that HWP and LWP athletes presented higher RPE values than LWE athletes after the first and third rounds. Nevertheless, LWE athletes presented greater RPE changes than HWE, HWP, and LWP athletes from the first to the second and third rounds. Therefore, these data suggest that MMA athletes competing at the elite level and lightweight class may perceive a fight as less demanding than professional athletes. On the other hand, it also indicates that LWE athletes present increased RPE values as the combat evolves. Most MMA fights are decided in the first (by a fight-ending sequence) or the last round (either by a fight-ending sequence or accumulated scorecards). This occurrence may help explain why professional athletes anticipate this with a higher RPE in the first and the last round, taking the second round as a recovery period ("cruising" is the common term used among coaches). Contrarily, less experienced athletes tend to display their larger output in the fists round (even if their RPE seems moderate) and then do not recover during the 1-min rest interval, thereby experiencing a larger and sudden rise in the RPE of the second round. Again, since this was one of the very few studies conducted in simulated MMA fights, this evidence still warrants confirmation.

These data corroborate with the results obtained by Milanez et al. (2011) in a study carried out with percussion athletes and research by Franchini et al. (2004) performed with judo athletes, where after simulations of three fights lasting 5 min, less experienced athletes showed a significant difference in RPE between the fights, considering the third fight as more strenuous. On the other hand, Siqueira et al. (2016) indicated values between 17 and 19 of the RPE after the end of a simulated fight, corresponding to very hard to maximal efforts. Furthermore, in the study by Amtmann et al. (2008), the authors observed values between 13 and 19 of the RPE after MMA fight simulations. The values in the present study were lower, which may be due to the combination of the simulated character of the combats with the high competitive level of the athletes.

### Limitations and Future Research

The current study presents several limitations we need to address. Firstly, the small sample size, which limits the statistical power and increases the type II error, does not enable us to generalize the results to other MMA athletes, and therefore they should be considered preliminary. Nevertheless, as previous Olympic coaches and researchers have mentioned, finding and recruiting a large and homogenous group of elite athletes of individual sports for research purposes is extraordinarily challenging and complex (Sands et al., 2005). Therefore, given the lack of studies on professional and elite MMA athletes, the current findings should be considered another step toward a better understanding of the technical and physical requirements of MMA fights according to the weight class and competitive level. Secondly, the simulated nature of the fights herein obviously impairs the athlete's full engagement and prevents them from applying some fight-ending sequences found during real competition. Therefore, it is suggested that future studies on the technical requirements should be based on real fighting analysis (probably through video records) and physiological measurements performed, whenever possible, during the fight itself (i.e., during the 1-min rest interval). Moreover, different athletes were allowed to use personal protection devices (i.e., helmets and shin guards, some variance could be due to different options by each athlete). Finally, future studies should also consider expanding the analysis of the physiological and psychophysiological responses during MMA fights by assessing the electrical activity in the brain, spinal cord, and muscles, as well as electrodermal activity as an indicator of arousal and stress (Bolach and Mickiewicz, 2019; Spanias et al., 2019). In addition to the detailed and objective data, these analyses can help understand the differences between professional and elite MMA athletes regarding the physiological and psychophysiological demands during MMA fights.

### Practical Implications

The results herein confirm that less experienced athletes may not be able to manage their effort (offensive action output), especially during the first round of a fight. Therefore, coaches are expected to avoid this tendency and attempt to find the optimal exertion that enables a good performance without exhausting the athlete in the early rounds.

## Conclusions

We conclude that lightweight athletes with less experience tend to start the fight with a higher technical output (more strikes) when compared with professional athletes. Furthermore, we also confirmed that lightweight athletes tended to start the first round with a lower heart rate when compared with heavyweight athletes, although the former can subsequently increase their heart rate throughout the remaining of the fight (as also shown by their RPE). Finally, we did not find differences between groups in blood lactate concentration and the readiness state.
